# Should We Subtype ADHD According to the Context in Which Symptoms Occur? Criterion Validity of Recognising Context-Based ADHD Presentations

**DOI:** 10.1007/s10578-018-0842-4

**Published:** 2018-08-30

**Authors:** Aja Louise Murray, Denis Ribeaud, Manuel Eisner, George Murray, Karen McKenzie

**Affiliations:** 1 Institute of Criminology, University of Cambridge, CB3 9DA Cambridge, UK; 2 Jacobs Center for Productive Youth Development, University of Zurich, Zurich, Switzerland; 3 Department of Psychology, Northumbria University, Newcastle, UK

**Keywords:** ADHD, Development, Informant discrepancies, Growth mixture modelling

## Abstract

ADHD symptoms show considerable individual variation in the contexts in which they are expressed. It has previously been proposed that subtyping individuals according to the contexts in which symptoms are expressed may be clinically useful. We examined context-based patterns of ADHD symptoms in a longitudinal cohort study of n = 1388 children, as well as context-specific and context-general predictors of symptoms. Participants were community-ascertained and provided ADHD symptom data at ages 7, 9, and 11. Using growth mixture modelling we identified five inattention and five hyperactivity/impulsivity categories that differed in the developmental patterns of symptoms reported by parent and teacher informants. We found some evidence that context-specific predictors were related to context-specific expressions. Specifically, after controlling for other risk factors for ADHD symptoms, relationships with teachers predicted school-specific (teacher-reported) but not home-specific (parent-reported) symptom levels. However, no subtypes defined by exclusively home-based symptoms emerged, suggesting that while symptoms may sometimes be specific to the school context, they are only rarely confined to the home context. Subtyping by context could be informative; however, further work will required to uncover the nature of any etiological, functional, or outcome differences between those who show symptom expression in different contexts.

**Electronic supplementary material:**

The online version of this article (10.1007/s10578-018-0842-4) contains supplementary material, which is available to authorized users.

## Introduction

Attention deficit hyperactivity disorder (ADHD) affects around 3.4% of children globally [1] and is characterised by pervasive and impairing levels of inattention and/or hyperactivity/impulsivity [2]. DSM-5 diagnostic criteria stipulate that for a diagnosis of ADHD to be given, problems must be evident across multiple contexts. However, a sizeable proportion of children show symptoms in only one context (or according to one informant) and may be no less impaired than children showing symptoms across multiple contexts. Furthermore, some authors have argued that it may be clinically useful to conceptualise children showing behavioural problems in specific contexts as representing distinct phenotypes [3]. The extent to which children can be meaningfully distinguished on the basis of the contexts in which they display ADHD symptoms has; however, yet to be established. In this study we thus evaluated the criterion validity of context-based presentation classifications (e.g. presentation at ‘home only’, ‘school only’, ‘both home and school’). We tested whether children differing in contexts of symptom expression differ in patterns of context-specific and context-general risk factors and sequalae.

DSM-5 diagnostic criteria for ADHD state that for a diagnosis ‘several inattentive or hyperactive-impulsive symptoms are present in two or more settings (e.g., at home, school, or work; with friends or relatives; in other activities)’ [2]. To determine if symptoms are present across contexts necessitates collecting information from more than one informant. For elementary school-aged children, this effectively means parents and teachers who can provide information on behaviour in the home and at school respectively. The ‘cross-context’ requirement of DSM-5 creates a challenge, however, because parents and teachers frequently disagree on the severity of ADHD symptoms displayed by a child. In a meta-analysis of multi-informant studies, for example, the average correlation between parent and teacher- reported ADHD was only .43 for inattention and .42 for hyperactivity/impulsivity [4], with studies published since broadly replicating these figures [5, 6]. The modest agreement between parents and teachers is not merely due to measurement error or informant biases (although both undoubtedly do contribute). Rather, evidence suggests that there are genuine differences in child behaviour across contexts/ in interaction with different informants. For example, informant unique perspectives on ADHD and related disruptive behaviour disorders show genetic influences, are stable over time, predict relevant outcomes, and can be mapped to differences in interactions with people who play different roles for the child in lab-based studies [7–10].

The most appropriate way to deal with contextual differences in ADHD symptoms in clinical practice, however, remains unclear. One suggestion is to conceptualise individuals with problems in different contexts according to different subtypes. Dirks et al. [3] argue that symptoms that occur in different contexts may constitute distinct phenotypes and that characterising these phenotypes has the potential to improve diagnosis and treatment. In this system, individuals with, for example, primarily school-based issues would be considered as a separate presentation from those with primarily home-based issues, who in turn would be considered a separate presentation from those with issues that spanned both contexts. ‘Subtyping’ of this kind is most likely to be clinically useful if individuals differing in the contexts in which their symptoms are expressed show distinct etiologies, prognoses, patterns of impairment, or treatment responses.

Evidence on the utility of distinguishing presentations on the basis of informant reports is, however, currently scant. A small number of studies have compared individuals with ‘pervasive’ ADHD, i.e. symptoms across multiple contexts to individuals who display problems in only a single context [10–14], with mixed results. While some studies have suggested that pervasive symptoms are associated with greater overall impairment [13, 14], others have found no difference between individuals with pervasive versus situation-specific symptoms [11]. One of the more recent studies to compare individuals differing in symptom contexts examined predictors of home- versus school-based problems as reported by parents and teachers respectively [10]. They found that a home-based risk factor (parental stress) predicted parent-reported symptoms only. They could not, however, rule out the possibility that this association reflected the response style of the parent because parents provided data on both constructs. They also found that parent-reported severity of symptoms was associated with symptoms across both contexts whereas teacher-reported severity was not. This hinted at the possibility that home-expressed symptoms are indicative of greater overall severity of problems than school-expressed symptoms.

In addition to parental stress, there are other ‘context-specific’ and ‘context-general’ factors that merit exploration to understand whether behavioural expression across contexts may be related to different etiologies and impairments. In the home, for example, negative parenting practices such as harsh or inconsistent discipline have been identified as important risk factors for, as well as outcomes of, disruptive behaviour disorders such as ADHD, conduct disorder and oppositional defiant disorder [15–17]. Analogous transactions may occur in the school environment where, for example, relationships with teachers can be affected by but can also shape disruptive behaviour problems [18–20]. Whether context-specific risk factors explain context-specific ADHD symptom expression is, however, not yet known.

In this study, we test the possibility that situation-specific problems have context-specific correlates using a large community-based longitudinal study. For comparison, we include a ‘trait-like’ predictor of ADHD: low self-control [21], which as a ‘trait’ is by definition are assumed to be expressed across multiple contexts. Using parent- and teacher- and self-reported data from the Zurich Project on Social Development from Childhood to Adulthood [z-proso; Eisner and Ribeaud 22] study, we use growth mixture modelling to first define subtypes of ADHD characterised by the contexts in which symptoms are evident and then assess whether these subtypes map to context-specific and context-general risk factors. This method allows the data to dictate categories defined by symptom trajectories over different contexts rather than imposing a priori classifications. Importantly, we use self-report measures of risk factors to ensure that any associations between risk factor and context do not simply reflect common rater bias. We focus not only on cross-sectional levels of symptoms, but patterns of symptom development over the elementary school years. This is based on past research suggesting considerable change in symptom levels over time is possible within individuals and that patterns of change meaningfully distinguish individuals [23]. We hypothesise that categories would emerge that represent unaffected individuals, individuals with home-specific presentation, individuals with school-specific presentation, and individuals with cross-context presentation. We also hypothesise that parenting would be particularly related to home-specific presentation and teacher relationships to school-specific presentation. However, we hypothesise that as a trait-like characteristic, self-control would not be differentially related to context-based presentation classifications.

## Method

### Participants

Participants were from the Zurich Project on Social Development from Childhood to Adulthood [z-proso; 22] cohort. Z-proso is an ongoing longitudinal study of development currently spanning ages 7–17. The current study concerns the measurement waves at age 7, 9 and 11. We focus on these waves because they are the waves at which data on child ADHD symptoms are available from both teacher and parent reports. The first measurement wave (age 7) took place in 2004. Sampling took place at the level of the school with a stratified random sampling procedure used to take account of school size and location. In each selected school (56 in total), all children who were due to enter the first grade in 2004 were invited to participate. The invitation was made via the parents of the target children, who provided consent on their behalf.

Of the baseline target sample size of n = 1675 children (all children entering first grade in the 56 selected schools), n = 1572 youth have contributed data for at least one wave of z-proso (94%). Data were available for n = 1388 (709 male) children in the current study (88% of the total recruited sample or 83% of the initial target sample). Children were included in the current study if ADHD data were available from at least one informant for at least one measurement wave. Previous analyses have evaluated whether, among those invited to participate in the study, those who declined to participate differed systematically from those who participated [24]. Predictors of participation that were tested included child gender, being in a special needs class, primary caregiver language, primary caregiver educational level, neighbourhood familialism, and neighbourhood social class. In bivariate analyses, social class, being in a small class, and some primary caregiver languages predicted non-response. The same study examined predictors of attrition over the years of the study. Predictors of attrition evaluated included the above-mentioned predictors, and parent- self- and teacher-reported behaviour: prosociality, ADHD symptoms, non-aggressive conduct problems, aggression, and internalising problems. In bivariate analyses, several behavioural dimensions significantly predicted drop-out in the waves included in the current study, including parent- but not teacher-reported ADHD symptoms. However, only primary caregiver language remained significant when including all predictors in a multiple regression. Given the overall pattern of results, considering the proportion of significant predictors and their effect sizes, the study concluded that the z-proso cohort can largely be considered representative of the same-aged underlying population, the main exception being that youth whose parents do not speak German (the official language of Zurich) as their first study are under-represented. Unfortunately, no data is available on why participants elected not to participate at baseline, or to drop-out. Eisner et al. [24], however, speculated that, because not speaking German as a first language is indicative of immigrant status, factors such as cultural differences, insecure residency status, and prior adverse experiences could have affected trust and willingness to participate. Our approach to dealing with non-random participation is discussed in the “Statistical Procedure” section.

The children included in the current study were of median age 7.03, 8.93, and 11.02 years of age at the three measurement waves. They came from a wide range of sociocultural backgrounds. Primary caregivers, for example, came from 70 different nations. Household socioeconomic status was available for n = 1097 of the children. Average International Socieconomic Index Scores [ISEI; 25] for this subsample was 48.9 (SD = 18.9). ISEI is metric of SES developed to provide a measure of occupational prestige that was internationally comparable. The average sample ISEI score of 48.9 corresponds to an occupational prestige level of a general manager in the wholesale and retail trade or a shop owner/manager [26]. The large standard deviation is indicative of the diversity of the sample in terms of SES.

### Measures

#### ADHD Symptoms

ADHD symptoms were measured using the *Social Behavior Questionnaire* [SBQ; 27] administered using the same items across parents and teachers. English translations of the items are provided in Table S1 of Supplementary Materials. Four items measure inattention and four measure hyperactivity/impulsivity. Responses are provided on a five-point Likert scale from *never* to *very often*. The reference period for the items is the past 6 months. Previous studies have provided evidence for the reliability, factorial validity, criterion validity, sensitivity to intervention effects, and developmental invariance of the ADHD SBQ items. This includes evidence from SBQ variants and translations administered across a number of child development studies internationally [27–30]. The psychometric properties of the SBQ ADHD items in the current sample have been explored in several previous publications, both directly in dedicated psychometric studies and indirectly in other empirical analyses [23, 31–34]. These previous studies have provided support for the reliability, developmental invariance, factorial validity, and criterion validity of the items across various waves of the z-proso study. As a measure of internal consistency in the current sample, omega reliability was calculated at each wave for parent and teacher reported inattention and hyperactivity/impulsivity. Unlike Cronbach’s alpha, omega does not involve the assumption of tau equivalence; an assumption that is very likely to be violated in practice [35, 36]. Values were all > .70 (ranging from .72 to .96) with the exception of parent-reported hyperactivity/impulsivity at age 7, which had an omega reliability of .65. Inattention and hyperactivity/impulsivity scores for each informant were obtained using a CFA analysis described in the “Statistical Procedure” section, where factor score determinacies are also reported. Descriptive statistics for the factor scores are provided in Table S2 of Supplementary Materials.

### School Functioning

Self-(child) reported current teacher and peer relationships at school were measured using 6 items measuring: *bond to teacher* and *bond to classmates*. Although academic functioning data were collected in z-proso, these were not included in the current study because these were teacher-reported (rather than objected test scores) and we wanted to focus on child-reported predictors to avoid inflated associations due to common rater bias. Children were asked to respond to the *bond to teacher* and *bond to classmates* items with respect to their current experiences. Responses were recorded on a 4-point Likert scale from *fully untrue* to *fully true*. We used the sum of the three items in each domain in the current study. A previous study in the current sample provided evidence for the reliability of the teacher relationships items [37]. Omega reliabilities were, in the current study, .79 for both *bond to teacher* and *bond to classmates*. The measures were developed specifically for the z-proso study and were selected after piloting in a previous Swiss sample. Some of the items were drawn from a large German comparative study on youth violence [38].

### Parenting

In terms of home environment, self-(child)reported *negative parenting* was measured using nine items which were adapted from the *Alabama Parenting Questionnaire* [*APQ*; 39] and the Parenting Scale from the Kriminologisches Forschungsinstitut Niedersachsen (KFN). These items measured erratic parenting, corporal punishment, and authoritarian parenting. Children were asked to respond with respect to their current experiences. Omega reliability was .71. Responses were provided on a four-point scale from *never* to *always*/*often*.

### Low Self-Control

Self(child)-reported *self-control* was measured using an adapted version of Grasmick’s [40] *Low self-control* questionnaire (subsequently modified by Longshore et al. [41]). The version administered at the age 11 wave of z-proso includes ten items measuring the domains of impulsivity, self-centredness, risk-seeking, volatile temper, and preference for physical over intellectual activities (Cronbach’s alpha = .75). Item contents are provided in Table S1 of Supplementary Materials. Children were asked to respond with respect to their current behaviour. Responses were provided on a four-point scale from *fully true* to *fully untrue*. The scale has been widely used in criminological research and is supported by a broad base of psychometric studies (e.g. see De Ridder et al. [42] for a review).

### Statistical Procedure

#### Growth Mixture Models for Inattention and Hyperactivity/Impulsivity

We used growth mixture models (GMMs) to summarise the heterogeneity in trajectories across individuals. We began by estimating factor scores for inattention and hyperactivity/impulsivity. Inattention and hyperactivity/impulsivity were modelled separately to reflect the evidence that they are dissociable cross-sectionally [43] and developmentally [23, 44]. For both, a longitudinal factor model was fit in which six latent inattention (or hyperactivity/impulsivity) factors were specified. These were two latent factors for each time point: one teacher-reported and one parent-reported. Each latent factor was defined by four indicators which were parallel across time and rater. All latent factors were allowed to correlate with one another. Residual covariances between the same items measured at different time points were also freely estimated. To achieve scaling and identification, the mean and variance of the parent-reported factors at age 7 were fit to 0 and 1 respectively. In addition, the loading and intercept of a reference indicator was constrained to equality across the six latent factors. Using the measurement models described above, factor scores were estimated, to be used in growth mixture model stages of analysis described below. The adequacy of factor scores was evaluated using model fit criteria and factor score determinancies. Measurement models were judged to show good fit if TLI and CFI were > .95, and RMSEA and SRMR were ≤ .05 [e.g. Hu and Bentler 45, 46]. Factor scores were considered adequate if determinacies were > .90 [47].

To account for selective drop-out by ADHD in the GMM models we used full information maximum likelihood estimation (FIML) and included all participants for whom at least one wave of data from at least one informant was available. FIML provides unbiased estimates provided that data are missing at random [MAR; 48]. MAR means that the data can be considered randomly missing, conditional on the predictors in the model. For the models predicting category membership, this method was not possible and listwise deletion was used. Listwise deletion only gives unbiased estimates when data are missing completely at random (MCAR), therefore, we can expect a small amount of bias from this method given that Eisner et al. [24] showed that teacher-reported ADHD symptoms were associated with drop-out (OR = 1.30).

The measurement model for inattention fit well by conventional criteria (CFI = .99, TLI = .99, RMSEA = .03, SRMR = .03) and yielded factor score determinacies ranging from .91 (parent-reported inattention at age 7) to .98 (teacher-reported inattention at all time points). The measurement model for hyperactivity/impulsivity showed acceptable fit by conventional criteria (CFI = .95, TLI = .93, RMSEA = .05, SRMR = .05) and yielded factor score determinacies ranging from .92 (parent-reported hyperactivity/impulsivity at age 11) to .99 (teacher-reported hyperactivity/impulsivity at age 9).

Using the inattention and hyperactivity/impulsivity factor scores calculated as described above, we evaluated models with between 1 and 7 classes, focusing on models with linear growth only (with only 3-time points, higher-order growth is not possible to model without the addition of further constraints). Growth was captured by intercept and slope factors, the variances and covariances of which were freely estimated within classes but fixed equal across classes. Time intervals were specified as proportional to the distances between the median sample ages at the 3-time points (t1 = 0, t2 = 0.42, t3 = 1). The median ages were derived from the full z-proso sample of n = 1572 for comparability with previous z-proso studies.

Model selection was based on the Lo-Mendall-Rubin [LMR; 49] test. The LMR test compares a k class model to a model with k-1 classes. A small *p* value (< .05) suggests that the former is a significantly better fitting than the latter. AIC, BIC and saBIC provide additional fit information with smaller (more negative) values suggesting that a model is better fitting. Where the LMR test provides an ambiguous result, information theoretic criteria can help with model selection.

### Predicting Class Membership from School, Home, and Cross-Situational Variables

Using the ‘best fitting’ growth mixture models for inattention and hyperactivity/impulsivity determined using the above-described procedure, multinomial regressions were used to predict category membership. A suitable reference category was chosen and the odds of being in each category, as compared to the reference category, were computed. To do this we used the three-step approach described by Asparouhov and Muthen [50]. In brief, a most likely class membership variable is created using the latent class posterior distribution from the growth mixture model estimation. This variable is regressed on the predictors and results are deattenuated for mis-classification uncertainty, also taken from the growth mixture model estimation. The advantage of this method is that predictors do not affect the formation of classes. We began by fitting unadjusted models with one predictor per model in order to estimate the bivariate associations between predictors and category membership. We then fit adjusted models including gender and all predictors in order to evaluate the unique effects of each predictor.

## Results

### Growth Mixture Models

#### Inattention

Fit statistics for the inattention growth mixture models with between 1 and 7 classes are provided in Table S3 of Supplementary Materials. An initial set of models encountered estimation problems which appeared to be due to a low within-class variance for teacher-reported slope factors. Fixing this variance to zero resolved the issue. The LMR test suggested that the 5-class solution was optimal.

**Fig. 1:**
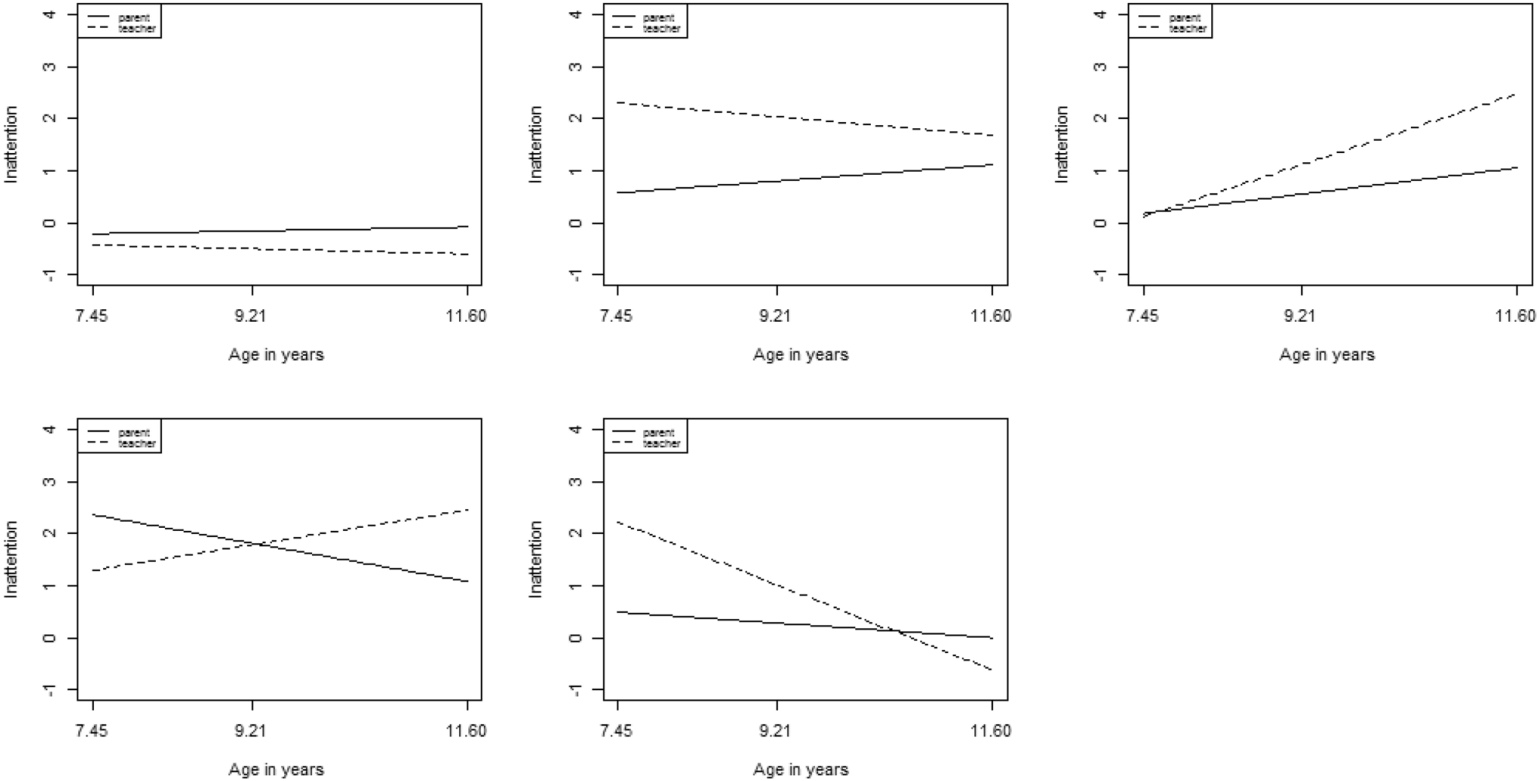
Growth trajectories for best fitting inattention GMM

Parameter estimates for the 5-class solution are provided in Table 1 and plotted in Fig. 1. These are based on unstandardized estimates and are thus on the scale of the inattention factor scores (see Table S4 in Supplementary Materials for inattention factor score descriptive statistics). The 5 classes were labelled ‘low stable’, ‘primarily school’, ‘increasing/primarily school’, ‘home → school’, and ‘decreasing/primarily school’. The ‘low stable’ class which accounted for 65% of the sample was characterised by low levels of inattention symptoms across the elementary school years, as reported by both parents and teachers. The ‘primarily school’ class (20% of the sample) was characterised by higher levels of symptoms reported by teachers than by parents, with the former reporting a slight decrease over time and the latter reporting a slight increase. The ‘increasing/primarily school’ class (8% of the sample) was characterised by increasing levels of inattention symptoms over the elementary school years but especially as reported by teachers. The ‘home → school’ class (1% of the sample) was characterised by decreasing parent-reported symptoms but increasing teacher-reported symptoms such that the context with the highest levels of reported symptoms switched from home to school over the elementary school years. The ‘decreasing/primarily school’ class (5% of the sample) initially showed high levels of symptoms as reported by teachers but these declined over the elementary school years.

**Table 1:** Parameters for best fitting inattention GMM

Class	Class label	Prevalence^a^	Parent intercept mean (SE)	Parent slope mean (SE)	Teacher intercept mean (SE)	Teacher Slope mean (SE)
1	‘Low stable’	.65	− 0.213 (0.03)	0.129 (0.03)	− 0.428 (0.06)	− 0.172 (0.06)
2	‘Primarily school’	.20	0.573 (0.08)	0.540 (0.05)	2.302 (0.10)	− 0.620 (0.10)
3	‘Increasing/primarily school’	.08	0.178 (0.10)	0.878 (0.09)	0.117 (0.12)	2.363 (0.16)
4	‘Home → school’	.01	2.363 (0.36)	− 1.280 (0.37)	1.297 (0.35)	1.156 (0.36)
5	‘Decreasing primarily school’	.05	0.490 (0.18)	− 0.490 (0.16)	2.226 (0.33)	− 2.840 (0.16)

^a^Based on estimated posterior probabilities

### Hyperactivity/Impulsivity

**Fig. 2:**
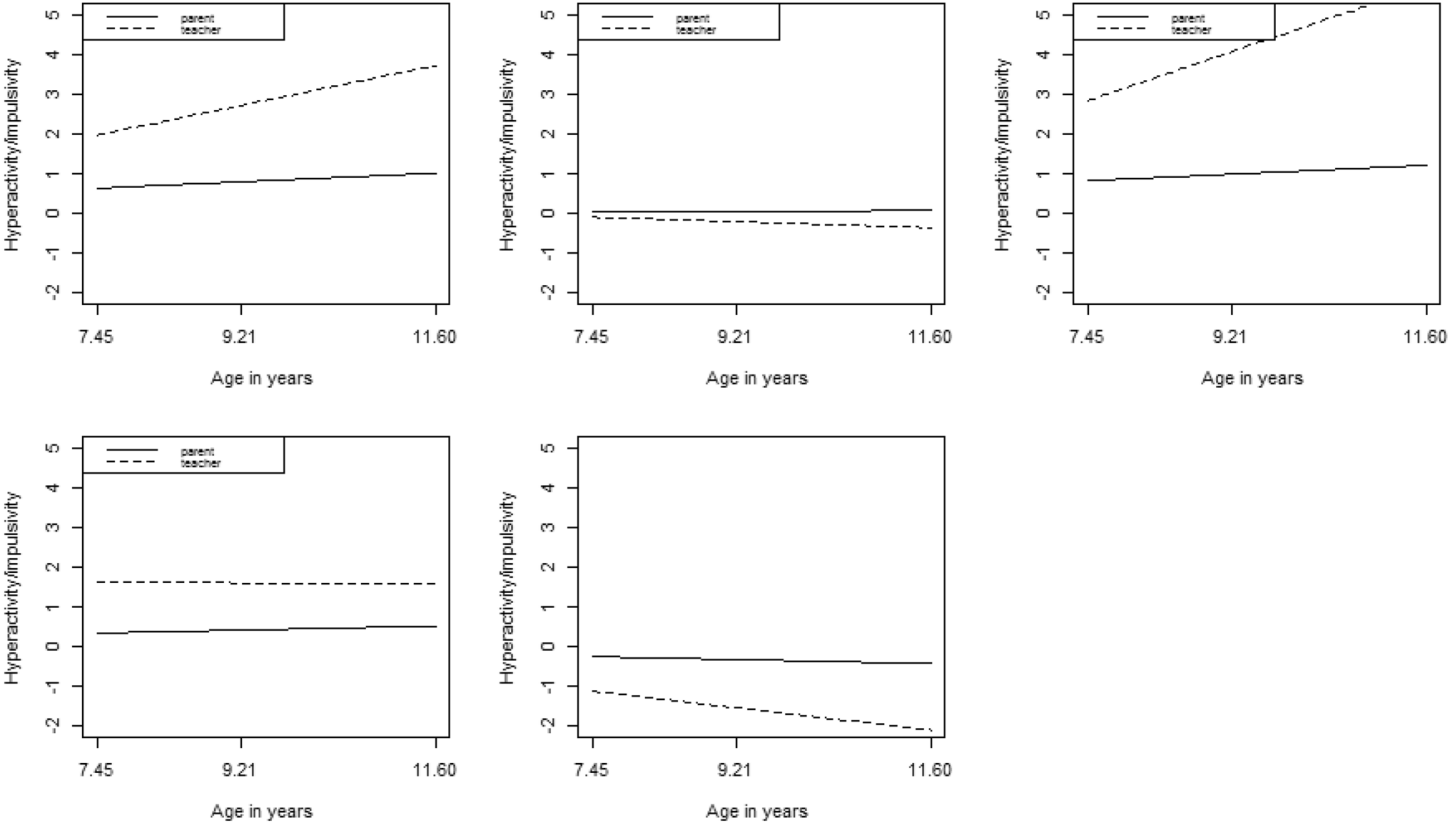
Growth trajectories for best fitting hyperactivity/impulsivity GMM

Fit statistics for the hyperactivity/impulsivity growth mixture models with between 1 and 7 classes are provided in Table S4 in Supplementary Materials. The LMR test suggested a 5-class solution was optimal. Parameter estimates for the 5-class solution are provided in Table 2 and plotted in Fig. 2. These are based on unstandardized estimates and are thus on the scale of the inattention factor scores (see Table S2 in Supplementary Materials for hyperactivity/impulsivity factor score descriptive statistics). The first class (accounting for 6% of the sample) was labelled ‘high increasing/primarily school’ and was characterised by high and escalating levels of symptoms as reported by the teacher but moderate and stable levels reported by the parent informant. The second class (27% of the sample) was labelled ‘moderate stable’ and was characterised by moderate symptom levels according to both informants across the elementary school years. The third class (3% of the sample) was labelled ‘very high increasing/primarily school’. It was similar to the ‘high increasing/primarily school’ but levels of hyperactivity/impulsivity were higher overall and increased faster according to teacher reports. The fourth class (15% of the sample) was labelled ‘high stable/primarily school’. This was characterised by moderate levels of symptoms reported by parents but high levels reported by teachers. The fifth class (49% of the sample), was labelled ‘low decreasing’. It was characterised by moderate levels of hyperactivity/impulsivity reported by parents but low and decreasing levels reported by teachers.

**Table 2:** Parameters for best fitting hyperactivity/impulsivity GMM

Class	Class label	Prevalence^a^	Parent intercept mean (SE)	Parent slope mean (SE)	Teacher intercept mean (SE)	Teacher slope mean (SE)
1	High increasing/primarily school	.06	0.637 (0.09)	0.383 (0.09)	1.979 (0.29)	1.753 (0.30)
2	Moderate stable	.27	0.046 (0.04)	0.028 (0.03)	− 0.094 (0.11)	− 0.262 (0.09)
3	Very high increasing/primarily school	.03	0.828 (0.14)	0.387 (0.10)	2.841 (0.36)	2.907 (0.34)
4	High stable/primarily school	.15	0.343 (0.06)	0.180 (0.04)	1.626 (0.63)	− 0.038 (0.17)
5	Low decreasing	.49	− 0.249 (0.03)	− 0.176 (0.02)	− 1.120 (0.06)	− 0.986 (0.06)

^a^Based on estimated posterior probabilities

### Predicting Class Membership

The results of the multinomial logistic regressions in which school, home and cross-situational factors predicted class membership for inattention are provided in Table 3 and for hyperactivity/impulsivity are provided in Table 4. Both unadjusted results, and results adjusted for gender and all other predictors are provided.

**Table 3:** Multinomial regressions for inattention

		Unadjusted			Adjusted		
		b	OR	*p*	B	OR	*p*
Class	Peers						
2	‘Primarily school’	− 0.12	0.89	.046	− 0.118	0.89	.086
3	‘Increasing/primarily school’	− 0.18	0.84	.003	− 0.045	0.96	.570
4	‘Home → school’	− 0.09	0.91	.370	− 0.056	0.95	.745
5	‘Decreasing/primarily school’	− 0.06	0.94	.524	− 0.082	0.92	.365

	Teacher						
2	‘Primarily school’	− 0.13	0.88	.027	− 0.025	0.98	.086
3	‘Increasing/primarily school’	− 0.298	0.74	< .001	− 0.218	0.80	.007*
4	‘Home → school’	− 0.077	0.93	.612	0.052	1.05	.840
5	‘Decreasing/primarily school’	0.114	1.12	.294	− 0.082	0.92	.365

	Parenting						
2	‘Primarily school’	0.080	1.08	.004	0.03	1.03	.337
3	‘Increasing/primarily school’	0.097	1.10	.002	0.06	1.06	.098
4	‘Home → school’	0.120	1.13	.020	0.103	1.11	.129
5	‘Decreasing/primarily school’	− 0.029	0.97	.500	− 0.046	0.96	.299

	Self-control						
2	‘Primarily school’	0.106	1.11	< .001	0.085	1.09	.004*
3	‘Increasing/primarily school’	0.099	1.10	.001	0.034	1.03	.316
4	‘Home → school’	0.038	1.04	.454	− 0.009	0.99	.862
5	‘Decreasing/primarily school’	− 0.024	0.98	.493	− 0.013	0.99	.722

**Table 4:** Multinomial regressions for hyperactivity/impulsivity

		Unadjusted			Adjusted		
		B	OR	*p*	B	OR	*p*
Class	Peers						
1	High increasing/primarily school	− 0.035	0.97	0.566	0.068	1.07	0.358
2	Moderate stable	− 0.047	0.95	0.303	− 0.044	0.96	0.382
3	Very high increasing/primarily school	− 0.197	0.82	0.013*	− 0.041	0.96	0.697
4	High stable/primarily school	− 0.138	0.87	0.010*	− 0.099	0.91	0.095

	Teacher					1.00	
1	High increasing/primarily school	− 0.210	0.81	0.001*	− 0.062	0.94	0.419
2	Moderate stable	− 0.075	0.93	0.096	− 0.054	0.95	0.293
3	Very high increasing/primarily school	− 0.296	0.74	< .001*	− 0.179	0.84	0.098
4	High stable/primarily school	− 0.143	0.87	0.010*	− 0.077	0.93	0.217

	Parenting					1.00	
1	High increasing/primarily school	0.077	1.08	0.014*	0.053	1.05	0.145
2	Moderate stable	0.008	1.01	0.700	0.099	1.10	0.990
3	Very high increasing/primarily school	0.134	1.14	< .001*	0.044	1.04	0.043*
4	High stable/primarily school	0.036	1.04	0.133	0.007	1.01	0.293

	Self-control					1.00	
1	High increasing/primarily school	0.127	1.14	< .001*	0.098	1.10	0.003*
2	Moderate stable	0.010	1.01	.568	− 0.001	1.00	0.974
3	Very high increasing/primarily school	0.114	1.12	.002*	0.044	1.04	0.293
4	High stable/primarily school	0.056	1.06	.007*	0.028	1.03	0.200

For inattention, there were a number of significant differences between the four classes showing some elevation in symptoms relative to the reference class (‘low stable’) in the bivariate analyses. However, there were only two significant ‘unique’ effects adjusting for gender and the other predictors. Specifically, having a poorer relationship with teachers significantly increased the odds of being in the ‘increasing/primarily school’ class while low self-control significantly increased the odds of being in the ‘primarily school’ class.

For hyperactivity/impulsivity, there were several significant differences between the reference class and the others; however, only two results remained significant in the analyses adjusting for genders and all other predictors. Specifically, parenting problems predicted membership in the ‘very high increasing/primarily school’ class and low self-control predicted membership in the ‘high increasing/primarily school’ class.

## Discussion

In this study, we sought to establish whether developmental subtypes of ADHD could be meaningfully distinguished on the basis of the contexts in which symptoms were primarily expressed. This builds on the idea proposed in previous research that distinguishing symptom presentations by informant could have clinical value [3]. Teachers served as informants for behaviour at school while parents served as informants for behaviour at home. For inattention, we found that if symptom reports varied across contexts, this was usually due to a greater expression of inattention at school. There was some evidence that school-based symptoms were related to school but not home problems, providing criterion validity support for informant-based presentation classification. For hyperactivity/impulsivity, children who showed elevated symptoms tended to show more severe school-based symptoms. However, differences in informant reports did not map to context-based predictors in the expected manner i.e., with school-based problems being particularly related to ADHD symptoms reported by teachers; home-based problems to symptoms reported by parents, and trait-like predictors to symptoms reported by both informants.

Our approach involved using growth mixture models to summarise classes of individuals with similar developmental trajectories and inattention and hyperactivity/impulsivity symptoms. Using this method, the majority of children were found to have low levels of ADHD by both informants. However, a pattern of informant discrepancy emerged, whereby when elevated symptoms were reported, teachers generally reported higher levels than parents. Inattention and hyperactivity/impulsivity have shown differing patterns of results in terms of informant discrepancy. For example, Murray et al. [5] found in a previous study in the current sample that while teachers tend to report higher levels of inattention on average, parents are more likely to report higher levels of hyperactivity/impulsivity. There is also evidence that inattention and hyperactivity/impulsivity differ in terms of their developmental trajectories. Using a similar technique to the current study, for example, Arnold et al. [44] found that in the *Longitudinal Assessment of Manic Symptoms* sample, developmental trajectories of inattention were best summarised in terms of three trajectories, while hyperactivity/impulsivity was best characterised in terms of four. We, therefore, analysed the dimensions of inattention and hyperactivity/impulsivity; discussed in turn below.

For inattention, five classes were judged optimal in the growth mixture analyses, four of which evidenced elevated levels of symptoms at some point according to at least one informant. Five classes is a larger number than those generally identified for either inattention or hyperactivity/impulsivity in previous studies of ADHD developmental trajectories [e.g. 23, 44, 51, 52]. Previous studies have, however, only included symptoms as reported by a single informant in their models and could, therefore, not identify distinctions between individuals with different patterns of expression across school and home contexts. The classes identified in the current study differed in overall levels of inattention symptoms as well as in developmental and informant pattern. Four of the classes evidenced informant discrepancies in levels and/or changes in inattention symptoms over time and two of these evidenced a ‘crossing-over’ effect whereby the informant who initially reported high levels reported lower levels by the end of the studied period, and vice versa.

The four classes characterised by elevated levels of inattention at any time across the elementary school years by either informant were compared to the class characterised by consistently low levels as reported by both informants. The classes were compared on ‘home’, ‘school’ and cross-situational inattention predictors. These predictors were reported by the child in order to avoid common rater bias. When considering potential predictors of class membership individually, issues with peers, teachers, parenting and low self-control all predicted membership in the classes characterised by consistently high or increasing levels relative to the ‘consistently low’ class. However, when considering the incremental contribution of the predictors (i.e., after controlling for gender and all other predictors), only two predictors were significant and both had modest effect sizes after adjustment for other predictors. Specifically, low self-control predicted membership in the class characterised by high stable levels (OR = 1.09) while teacher problems predicted membership in the class characterised by increasing levels of symptoms (OR = 0.80).

These results support the idea that consistently high levels of symptoms may be predicted by individual ‘trait-like’ features of the child such as low self-control, whereas changing levels may derive from the onset of time-varying influences such as poor relationships with teachers. In addition, the fact that teacher but not parent factors uniquely predicted symptoms that were particularly high at school supports the criterion validity of the school-specific inattention subtype.

An analogous set of analyses were conducted for hyperactivity/impulsivity symptoms. Here, five classes were also judged optimal, four of which evidenced a discrepancy between informants. When comparing these five classes on their levels of home, school and cross-situational correlates, the class with the lowest overall levels of hyperactivity/impulsivity symptoms (in which parents reported higher levels than teachers) served as the reference category. Bivariate analyses suggested that other than the ‘moderate stable’ class, classes could generally be differentiated from the reference class on the basis of peer, parent, and teacher problems and on low self-control. Examining the unique contributions of these predictors, however, there were only two significant results, both again with modest effect sizes after controlling for other predictors. First, parenting problems predicted membership in the ‘very high increasing/primarily school’ (OR = 1.04) class while low self-control predicted membership in the ‘high increasing/ primarily school’ (OR = 1.10) category. These two classes were the most ‘severe’ classes, i.e. they appeared to show the highest overall levels of symptoms and, therefore, it is not surprising that where ADHD risk factors uniquely significantly predicted class membership, it was in these two classes. This is all the more so given that both self-control and parenting were measured at age 11, the point on these two trajectories where hyperactivity/impulsivity symptoms were at their peak. It is less clear why low self-control predicted membership in the second most severe class but not the most severe class. Possibly those with the highest levels of ADHD symptoms are poorer at accurately recognising deficits in self-control, consistent with the positive illusory bias that has been observed in youth with ADHD [53]. However, these results don’t support the hypothesised mappings of home, school and cross-context risk factors with ADHD symptom presentations in the corresponding contexts. Rather, they suggest that for hyperactivity/impulsivity, risk factors in the home are related to symptoms at school over and child low self-control and relationships with teachers.

Taken together and as anticipated, our results suggested different patterns for inattention and hyperactivity/impulsivity. Although both could be summarised in terms of five trajectory classes, the trajectories represented in the classes differed. For example, while hyperactivity/impulsivity tended to be stable across development in the home context, evidencing variability mainly at school, inattention showed changes over development in both contexts. In addition, while there were two inattention classes in which the context of greatest severity swapped over time, the context of greatest severity remained constant across development for all of the hyperactivity/impulsivity classes. Further, while the inattention classes could to some degree be mapped to context-specific and context-general predictors, the same could not be said for hyperactivity/impulsivity. Arguably this suggests that utilising specifiers to indicate the context of greatest symptom expression could be more informative for inattention than for hyperactivity/impulsivity.

While it would be premature to derive any clinical implications from the current study, our results are indicative of the potential utility of further exploring the introduction of ADHD presentations based on the context(s) in which symptoms are expressed. This would represent a more nuanced approach than the current situation in which an individual must show significant symptoms across multiple domains to receive a diagnosis. This means that individuals with severe symptoms could be missing out on support and interventions from which they could benefit if clinically significant symptoms cannot be evidenced across multiple contexts. An alternative proposal would be to utilise a single cut-off for severity but to use a specifier to identify the primary contexts in which symptoms are present. Several steps will be required to evaluate the potential clinical utility of this approach. First, the mixture analyses of the current study should be replicated in other datasets to establish which context-based presentation categories are replicable. Second, a broader range of risk factors should be analysed to assess whether individuals with presentations in different contexts (and multiple versus single contexts) appear to differ in etiology. Third, it should be evaluated whether these presentations are associated with different or more severe patterns of psychosocial impairment. For example, whether those with symptoms across multiple contexts are more prone to common ADHD comorbidities such as anxiety and depression, oppositional defiant disorder, and conduct disorder. Where possible it should be evaluated whether presentations in different contexts are related to treatment responses. For example, teacher- and parent-administered interventions are recommended as psychosocial treatments for ADHD [54]; however, the extent to which an individual benefits from one or the other could depend on whether their symptoms are more severe in the context of school or home. Finally, though our focus was on ADHD symptoms, similar differences in symptoms of related disorders may also be expected to show meaningful subtypes according to contexts or situations [55]. Thus, it would be of interest to replicate the current study with oppositional and conduct problems.

It is important to note the limitations of the current study. Though our results suggested only limited evidence for the criterion validity of informant-based developmental subtypes, it included only a handful of home- school- and cross-situational predictors and it would be beneficial to explore associations with a broader range of established context-specific and context-general ADHD predictors in future research. In addition, two of our criterion measures (teacher relationships, peer relationships), though evidencing good reliability in the current study, have undergone limited prior psychometric evaluation. More broadly, concerns are sometimes raised about the validity of self-reports at younger ages. Our parenting measure showed a slightly low reliability, only just exceeding conventionally accepted levels (Omega = .71), suggesting that its associations with trajectory classes could have been under-estimated due to reliability attenuation. However, while there is evidence that measurement error is slightly greater in the age 11 reports, there is little evidence in z-proso that the age 11 self-reports are substantially less reliable or valid than self-reports taken at ages 13,15 or 17 in general [31, 56]. Separately, however, it has been noted that individuals high in ADHD traits may have difficulty accurately reporting on their symptoms [57]. Logically this difficulty could extend to self-reports of other constructs and differentially affect criterion associations with membership in the various ADHD symptom presentation classes identified in the current study. Nevertheless, concerns about utilising self-reports for the criterion variables must be weighed against the fact that utilising parent- or teacher- reports for these could inflate associations due to common rater bias. This issue could be addressed in future studies using an additional independent informant and behavioural measures of self-control.

A second limitation of our study was the brevity of our ADHD measure; a function of being administered as part of a large cohort study. Replication with more comprehensive ADHD measures would be valuable. Second, as discussed in the introduction, differences in informant reports are not entirely due to differences in child behaviour. Measurement error and informant biases also play a role. Future studies that can control for informant characteristics such as stress or mental health problems, especially depression [58, 59] can better isolate context-differences that are related to the child behaviour specifically.

Finally, for our analyses predicting category membership, FIML was not available and, therefore, listwise deletion was used. These analyses could, therefore, have been affected by non-random non-response. Given the patterns of non-random non-response identified by Eisner et al. [24], the most likely impact of this is an attenuation of the associations between predictors and ADHD class membership.

### Summary

Our study found some support for subtyping ADHD symptoms on the basis of the informants who provide the information about symptoms. Growth mixture analyses in a normative sample of 1388 youth identified five categories that were that were distinguishable on the basis of informant reports of developmental trajectories. These categories included presentations in which symptoms were particularly elevated in school relative to home but none where they were particularly elevated at home compared to school. This suggests that were context-specific presentations occur, severity is more likely to be greater at school than at home. The categories identified showed only a weak tendency to map to context-specific and context-general predictors of ADHD. One exception was the finding that teacher-relationships uniquely and specifically predicted a rapid increase in inattention symptoms at school only. On balance, our results point to potential value in further exploring presentations that differ according to context. In particular, future studies could evaluate whether similar categories emerge in different samples and whether they can be mapped to etiological, functional, and outcome differences.

## Electronic supplementary material

Below is the link to the electronic supplementary material.

Supplementary material 1 (DOCX 23 KB)
