# Supplementary material for: Should We Subtype ADHD According to the Context in Which Symptoms Occur? Criterion Validity of Recognising Context-Based ADHD Presentations
Source: Child Psychiatry Hum Dev. 2018 Aug 30;50(2):308–20. doi: 10.1007/s10578-018-0842-4 (PMC6428792; doi:10.1007/s10578-018-0842-4)
Supplement: Supplementary file 1 — Supplementary material 1 (DOCX 23 KB) [file 10578_2018_842_MOESM1_ESM.docx]

**Supplementary Materials**

**Table S1: Questionnaire items translated and paraphrased**

| **Domain** | **Item content** | **Informant(s)** |
| --- | --- | --- |
| **Social Behaviour Questionnaire** | | |
| **Inattention** | <CHILD> cannot settle to anything for more than a few moments. | Parent, teacher |
| **Inattention** | <CHILD> is distractible, has trouble sticking to any activity. | Parent, teacher |
| **Inattention** | <CHILD> can't concentrate, can't pay attention for long. | Parent, teacher |
| **Inattention** | <CHILD> is inattentive. | Parent, teacher |
| **Hyperactivity/impulsivity** | <CHILD> is impulsive, acts without thinking. | Parent, teacher |
| **Hyperactivity/impulsivity** | <CHILD> has difficulty awaiting turn in games or groups. | Parent, teacher |
| **Hyperactivity/impulsivity** | <CHILD> can't sit still, is restless, or hyperactive. | Parent, teacher |
| **Hyperactivity/impulsivity** | <CHILD> fidgets. | Parent, teacher |
| **Teacher relationships** | | |
| **Teacher bond** | My teacher treats me fairly. | Child |
| **Teacher bond** | My teacher helps me when necessary. | Child |
| **Teacher bond** | I get on well with my teacher. | Child |
| **Peer relationships** | | |
| **Peer bond** | I get on well with the other kids in my class. | Child |
| **Peer bond** | We have a really good sense of community within the class. | Child |
| **Peer bond** | The other kids in my class are nice to me. | Child |
| **Negative parenting** | | |
| **Corporal punishment** | Your parents slap you as a disciplinary measure. | Child |
| **Corporal punishment** | Your parents spank you with their hand as a disciplinary measure. | Child |
| **Corporal punishment** | Your parents pull your ears or hair as a disciplinary measure. | Child |
| **Erratic** | Your parents threaten to punish you but don’t follow through. | Child |
| **Erratic** | Your parents take away privileges or money as punishment. | Child |
| **Erratic** | Your parents punish you more severely when they are in a bad mood. | Child |
| **Authoritarian** | Your parents are very strict with you when you don’t do exactly as they say. | Child |
| **Authoritarian** | Your parents order you around and do not let you talk back to them. | Child |
| **Authoritarian** | Your parents show you that they are in charge. | Child |
| **Low self-control** | | |
| **Impulsive** | Acting spontaneously, without thinking. | Child |
| **Self-centred** | Getting what I want. | Child |
| **Risk** | Taking risks for fun. | Child |
| **Temper** | Getting mad when not getting something. | Child |
| **Physical** | Action rather than thinking. | Child |
| **Self-centred** | Don’t care if upset others. | Child |
| **Temper** | Lose control easily. | Child |
| **Impulsive** | Preference for physical over intellectual. | Child |
| **Impulsive** | Act without considering consequences. | Child |
| **Risk** | Action better than security. | Child |

**Table S2: Descriptive statistics for ADHD factor scores**

|  | **n** | **Mean** | **SD** | **Min** | **Max** | **Range** |
| --- | --- | --- | --- | --- | --- | --- |
| **Inattention** | | | | | | |
| **Parent report age 7** | 1388 | 0.00 | 0.87 | -1.43 | 3.50 | 4.93 |
| **Parent report age 8** | 1388 | 0.37 | 0.98 | -1.36 | 3.85 | 5.21 |
| **Parent report age 11** | 1388 | 0.26 | 1.04 | -1.56 | 4.04 | 5.59 |
| **Teacher report age 7** | 1388 | 0.43 | 1.61 | -1.63 | 4.36 | 6.00 |
| **Teacher report age 8** | 1388 | 0.16 | 1.55 | -1.67 | 4.34 | 6.01 |
| **Teacher report age 11** | 1388 | 0.24 | 1.59 | -1.74 | 4.41 | 6.15 |
| **Hyperactivity/impulsivity** | | | | | | |
| **Parent report age 7** | 1388 | 0.00 | 0.85 | -1.37 | 2.72 | 4.09 |
| **Parent report age 8** | 1388 | 0.07 | 1.03 | -1.62 | 3.53 | 5.15 |
| **Parent report age 11** | 1388 | -0.02 | 1.09 | -1.73 | 3.72 | 5.45 |
| **Teacher report age 7** | 1388 | -0.08 | 2.24 | -2.51 | 5.93 | 8.44 |
| **Teacher report age 8** | 1388 | -0.55 | 2.16 | -2.68 | 5.91 | 8.59 |
| **Teacher report age 11** | 1388 | -0.50 | 2.08 | -2.55 | 6.26 | 8.80 |

**Table S3: Model fits for inattention GMMs**

| **Number of classes** | **AIC** | **BIC** | **saBIC** | **Entropy** | **LMR** | **LMR *p*-value** |
| --- | --- | --- | --- | --- | --- | --- |
| **1** | 23018.21 | 23101.98 | 23051.16 | - | - | - |
| **2** | 22833.65 | 22943.59 | 22876.89 | 0.75 | 189.33 | 0.00 |
| **3** | 22705.73 | 22841.85 | 22759.26 | 0.77 | 134.21 | 0.00 |
| **4** | 22622.98 | 22785.29 | 22686.81 | 0.79 | 90.25 | 0.02 |
| **5** | 22579.08 | 22767.56 | 22653.20 | 0.82 | 52.45 | 0.02 |
| **6** | 22543.80 | 22758.46 | 22628.22 | 0.82 | 44.06 | 0.47 |
| **7** | 22502.74 | 22743.58 | 22597.46 | 0.82 | 49.69 | 0.02 |

**Table S4: Model fits for hyperactivity/impulsivity GMMs**

| **Number of classes** | **AIC** | **BIC** | **saBIC** | **Entropy** | **LMR** | ***p*-value** | |
| --- | --- | --- | --- | --- | --- | --- | --- |
| **1** | 23613.05 | 23707.29 | 23650.11 | NA | NA | - |  |
| **2** | 23218.76 | 23339.17 | 23266.11 | 0.87 | 393.42 | <.001 |  |
| **3** | 23009.98 | 23156.57 | 23067.63 | 0.88 | 212.89 | 0.001 |  |
| **4** | 22912.68 | 23085.46 | 22980.63 | 0.87 | 104.41 | .0024 |  |
| **5** | 22515.59 | 22714.54 | 22593.83 | 0.96 | 313.97 | <.001 |  |
| **6** | 22477.71 | 22702.84 | 22566.24 | 0.94 | 46.60 | 0.2405 |  |
| **7** | 22403.59 | 22654.90 | 22502.42 | 0.93 | 5.77 | 0.7097 | |
